# Microbiome Composition in Microscopic Colitis: A Systematic Review

**DOI:** 10.3390/ijms24087026

**Published:** 2023-04-10

**Authors:** Aleksandra Garczyk, Marcin Mardas, Marta Stelmach-Mardas

**Affiliations:** 1 Department of Obesity Treatment, Metabolic Disorders and Clinical Dietetics, Poznan University of Medical Sciences, Szamarzewskiego Street 84, 60-569 Poznan, Poland; 2 Department of Gynaecological Oncology, Institute of Oncology, Poznan University of Medical Sciences, 60-569 Poznan, Poland; marcin.mardas@ump.edu.pl

**Keywords:** microscopic colitis, collagenous colitis, lymphocytic colitis, microbiome, microbiota, intestinal flora, systematic review

## Abstract

Believed to be a rare cause of chronic diarrhoea, microscopic colitis (MC) is a condition with rising incidence. Many prevalent risk factors and the unknown pathogenesis of MC rationalise the need for studies on microbiota composition. PubMed, Scopus, Web of Science and Embase were searched. Eight case-control studies were included. The risk of bias was assessed with the Newcastle–Ottawa Scale. Clinical details on the study population and MC were poor. The most consistent result among the studies was a decreased *Akkermansia* genus in faecal samples. Other results were inconsistent due to the different taxonomic levels of the outcomes. Possible changes in different taxa were observed in patients who suffered from MC compared to healthy controls. The alpha diversity compared between MC and the diarrhoea control may suggest potential similarities. The beta diversity in MC compared to healthy and diarrhoeal populations showed no significant outcomes. The microbiome composition in MC possibly differed from the healthy control, but no agreement regarding taxa was made. It might be relevant to focus on possible factors influencing the microbiome composition and its relationship with other diarrhoeal diseases.

## 1. Introduction

Chronic watery diarrhoea is the predominant demonstration of microscopic colitis (MC) [1,2]. Other associated symptoms are faecal urgency, nocturnal diarrhoea and faecal incontinence [2]. A diagnosis of MC requires a colonic biopsy, which might also reveal subtypes of MC: lymphocytic colitis (LC), collagenous colitis (CC) [1,2] or an incomplete form [2]. There is no evidence suggesting that MC might be connected with a higher risk of colorectal cancer [1,2], adenoma [2] or surgery [1]. Nevertheless, it might have a detrimental effect on patients’ quality of life [1,2].

MC is sometimes believed to be a rare inflammatory bowel disease (IBD). However, the incidence of MC in Western countries has increased and is estimated at 11.4 per 100,000 person-years, with a prevalence of 119 per 100,000 persons [2], ranging from 48 to 219 per 100,000 [1]. Moreover, it occurs in 7.5% of patients who are diagnosed with chronic diarrhoea [1] and might be responsible for 12.8% of unexplained cases (high heterogeneity data) [2]. There are few risk factors associated with the development of MC, such as current and former smoking, the use of proton pump inhibitors (PPIs), nonsteroidal anti-inflammatory drugs (NSAIDs) or selective serotonin reuptake inhibitors (SSRIs) [2]. Furthermore, women are more likely to suffer from MC than men [1,2]. There are many possible factors contributing to the nonestablished pathogenesis of MC, and those that have been considered are luminal factors, extracellular matrix remodelling, involvement of the immune system and genetic predispositions [2,3]. One presumed luminal factor is microbiome composition, the alteration of which might occur in MC. Importantly, changes in the microbiome composition have been recognised in Crohn’s disease (CD) and ulcerative colitis (UC) [4], highlighting the rationale behind microbiome analysis in MC.

Not only the pathogenesis but also the treatment of MC have not yet been firmly determined. Currently, the first-choice of treatment is budesonide. It was proven to be effective and safe in MC [1,2]. The response rate for budesonide treatment was 81% for collagenous colitis (CC) and 84% for lymphocytic colitis (LC) [2], while one-third of patients remained free of symptoms [1]. Treatment for nonresponders might vary. Some possibilities are available, but no strong recommendation can be made [2]. Additionally, the maintenance strategy for MC is not yet fully determined. There is evidence in favour of budesonide therapy [1,2]. One possible treatment in the future may be the administration of probiotics. However, uncertainties concerning the mechanisms of probiotics’ action and efficacy still remain [5]. Nevertheless, they are considered safe agents [6], and acknowledging whether the microbiome plays a role in the pathogenesis of MC might put probiotics use into perspective.

Based on the available data, we decided to cover the aspect of microbiota composition in microscopic colitis to provide practical insight into the further guidance of patients. The microbiota composition in microscopic colitis has not yet been fully determined. A new understanding of microbiota’s role in the pathogenesis of MC might open the door for personalised treatment.

## 2. Materials and Methods

### 2.1. Search Strategy

This review was based on a search of PubMed, Scopus, Web of Science and Embase to assess studies on the microbiome. The search strategies were adapted for each of the databases: MeSH terms for the PubMed search and Emtree terms for Embase. Adjustments for Scopus and Web of Science, the following were included:

*#1 Microscopic Colitis OR Colitis, Microscopic OR Lymphocytic Colitis OR Colitis, Lymphocytic OR Colitis, Collagenous OR Collagenous Colitis*

*#2 Microbiota OR Microbial Community OR Community, Microbial OR Microbial Community Composition OR Community Composition, Microbial OR Composition, Microbial Community OR Microbial Community Structure OR Community Structure, Microbial OR Microbial Community Structures OR Microbiome OR Human Microbiome OR Microbiome, Human OR Gastrointestinal Microbiome OR Microbiome, Gastrointestinal OR Gut Microbiome OR Microbiome, Gut OR Gut Microflora OR Microflora, Gut OR Gut Microbiota OR Microbiota, Gut OR Gastrointestinal Flora OR Flora, Gastrointestinal OR Gut Flora OR Flora, Gut OR Gastrointestinal Microbiota OR Microbiota, Gastrointestinal OR Gastrointestinal Microbial Community OR Microbial Community, Gastrointestinal OR Gastrointestinal Microflora OR Microflora, Gastrointestinal OR Gastric Microbiome OR Microbiome, Gastric OR Intestinal Microbiome OR Microbiome, Intestinal OR Intestinal Microbiota OR Microbiota, Intestinal OR Intestinal Microflora OR Microflora, Intestinal OR Intestinal Flora OR Enteric Bacteria OR Flora, Intestinal OR Bacteria, Enteric OR Microbiotas OR Microbial Communities OR Microbial Community Compositions OR Microbiomes OR Human Microbiomes OR Gastrointestinal Microbiomes OR Gut Microbiomes OR Gut Microbiotas OR Gastrointestinal Microbiotas OR Gastrointestinal Microbial Communities OR Intestinal Microbiomes OR Gastric Microbiomes OR Intestinal Microbiotas OR alimentary canal flora OR alimentary tract flora OR bowel flora OR bowel microbiota OR digestive canal flora OR digestive tract flora OR enteric flora OR enteric microbiota OR flora, intestine OR gastrointestinal canal flora OR gastrointestinal tract flora OR gastrointestine flora OR gastrointestine tract flora OR gut bacteria OR intestinal bacteria OR intestinal bacterial flora OR intestinal bacterium OR intestinal canal flora OR intestinal microbe OR intestinal microbes OR intestinal microorganism OR intestinal tract flora OR intestine bacteria OR intestine bacteria change OR intestine bacterial flora OR intestine bacterium OR intestine microbial flora OR bacterial biome OR bacteriobiome OR bacteriome OR bacterial microbiome OR microflora OR intestine microflora OR feces microflora*

*Search: #1 AND #2*

The results of the search of the databases were acquired on 19 January 2023 for microbiome-related studies. No database filters were used.

### 2.2. Study Selection

**Figure 1:**
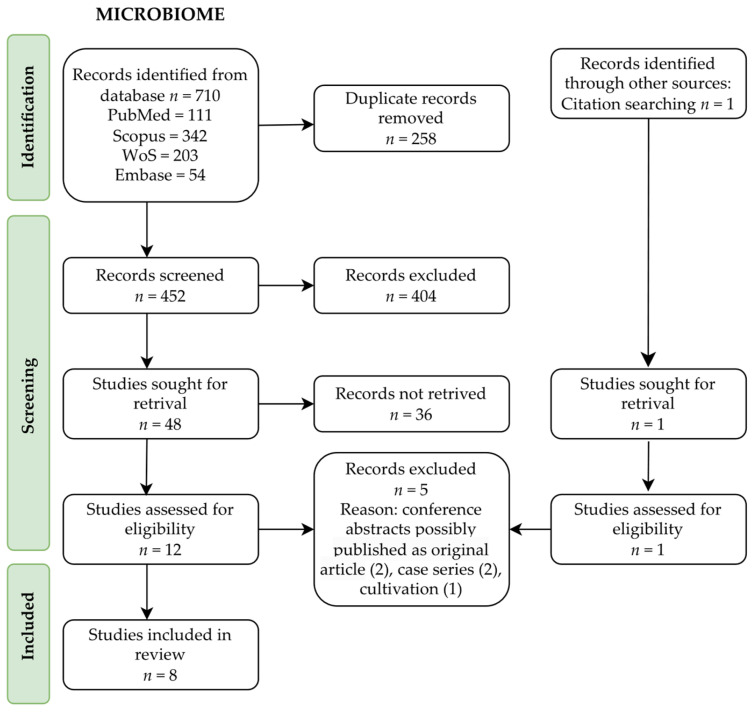
Flow diagram regarding microbiome-related studies.

The results of the conducted search are presented as a flow diagram according to the PRISMA statement (Figure 1). All of the results were assessed by two researchers. First, the duplicates were removed with the help of Zotero software. Second, the titles were screened to choose articles possibly related to the topic. Studies in English or German were included. The records regarding paediatric populations and the assessment of the microbiome by cultivation were excluded. Any doubt regarding the article’s title was an indication to include it in the next step. Finally, the abstracts were screened, and the articles were chosen for a full-text assessment. During this phase, case reports and case series were excluded. For occurring doubts concerning the inclusion of an article, the other authors were consulted. 

### 2.3. Data Extraction and Analysis

All articles that were assessed as eligible for this review were screened for the study’s characteristics, population description, features of MC and associated diarrhoea, microbiome composition based on sequencing and the alpha and beta diversity indices. The derived details were double-checked for mistakes. Any doubts were dispelled by the co-authors or described in the study. Whenever needed, the authors of the included articles were contacted by email in order to ask about details. 

Due to the heterogeneity of the data regarding the microbiome, a meta-analysis was not performed. Therefore, only a narrative characterisation of the outcomes was included in the present study.

### 2.4. Risk of Bias

The quality of the studies was assessed independently for the microbiome using the Newcastle–Ottawa Quality Assessment Scale for case-control studies (CCS). A full score using this scale is 10 points. A study with ≥6 points was regarded as a good-quality study.

## 3. Results

### 3.1. Description of Study Design and Population

The performed selection of studies resulted in the inclusion of eight articles regarding microbiome composition [7,8,9,10,11,12,13,14] (Table 1). Every included study was conducted in Western countries and designed as a CCS.

First, an analysis and description of the study population were performed. The study population examined for the microbiome composition was *n* = 183. Most of the studies assessed the microbiome composition without a targeted intervention and compared it to the control group [7,10,11,13,14]. Different control groups were applied in separate studies. Healthy controls were the most prevalent and consisted of *n* = 121. Other control groups regarded diseases associated with diarrhoea, including bile acid diarrhoea (*n* = 16) [9], functional diarrhoea (*n* = 31) [9,14], Crohn’s disease (*n* = 53) [10,11] and ulcerative colitis (*n* = 70) [10,11]. In [7], the control group was described as patients reporting loose stools (*n* = 153). The overall description of the clinical details of MC was poor (Table 2). Each study was conducted in an adult population only. Women were more prevalent in seven out of eight studies, except the study by Millien et al. [8]. Only four studies reported body mass index (BMI), indicating a wide range of patient inclusion (underweight, overweight and obese) [7,9,11,14]. Autoimmune disorders and budesonide treatment were reported in one study only [11]. Additionally, each patient in the study by Krogsgaard et al. [12] was treated with budesonide after the first microbiome assessment. Few studies reported the prevalence of risk factors among the participants. Each of them might act as an agent, which influences the microbiome composition. Current and former smoking were indicated in four studies [8,12,13,14]. The only reported drugs taken were proton pump inhibitors (PPIs) [8,12] and nonsteroidal anti-inflammatory drugs (NSAIDs) [8,9]. The elimination of the use of antibiotics before inclusion was mandatory in six studies: 3 months [7,8,9,11,12] and 1 month [10]. Probiotics were eliminated for 3 months [9] or 1 month [12].

### 3.2. Details about Microscopic Colitis

The following clinical details about the MC course were described in the included studies (Table 3.). The subtype was defined in four out of eight studies. One study included patients diagnosed with collagenous colitis only [11]. Currently, there are no differences in the clinical management for any of the subtypes [1]. However, some diversity in the evidence for LC and CC occurred [2]. Three studies verified the diagnosis of MC through biopsy before inclusion [7,8,9]. Regarding the clinical activity of MC, it should be assessed using the Hjorstwang criteria [15]. However, they were applied only by Carstens et al. [11] and Krogsgaard et al. [12]. The duration of diarrhoea was assessed in one study, with a median of 20 weeks [9]. In the study by Milien et al. [8], 40% of patients had diarrhoea for less than 12 months. Daily bowel movements were described in two studies [8,9].

### 3.3. Microbiome Composition Analysis

The results of the microbiome assessment differed among the studies (Table 4). Biopsy [7,8] or faecal samples [9,10,11,12,13,14] were obtained. The method of the microbiome analysis was based on sequencing technology. Most of the studies applying sequencing methods relayed 16s RNA [7,8,9,10,11] or 16s DNA [12]. However, two studies were based on metagenomic sequencing [13,14]. The outcomes of the alpha diversity assessment for MC and healthy controls showed no differences in three studies [8,9,11]. However, in [10,12] the alpha diversity was significantly lower in MC than in the healthy control. One study [12] proved that the alpha diversity was higher in MC after treatment with budesonide than at the beginning. Differences were not detected for the beta diversity [8]. The microbial dysbiosis index was noticed to be higher in active MC than in the remission phase [14]. Additionally, there were no differences observed between LC and CC in the alpha diversity [9,10], beta diversity [9], microbial dysbiosis index [9] and bacterial abundance [12]. Second, the differences between MC and other diarrhoea controls (DCs) were checked. There were no differences in the alpha diversity between MC and CD or UC [10] or regarding the functional DC and bile acid DC [9]. In one study, the alpha diversity for MC, functional DC and bile acid DC together was lower than for HC [9]. The microbial dysbiosis index was also calculated for the DC and was proved to be higher in the active phase of MC than in the functional DC [14] and higher in MC than in the functional DC together with the bile acid DC [9]. These results indicate that there might be some differences between MC and HC. Moreover, there is the possibility that there were some similarities between MC and the DC, or another factor, in common that influenced the results.

The outcomes of the detailed microbiome composition were presented as increased or decreased taxa in MC compared to the healthy control. Most of the studies used a level of significance of <0.05 [9,11,12,13]. Except for [13], all studies reported adjusting results with a correction for multiple testing. All studies used different taxonomic levels in their reports. Therefore, the reported taxa were analysed for their taxonomic levels to assess whether patterns among the described taxa occurred. For this purpose, the National Center for Biotechnology Information (NCBI) Taxonomy’s browser [16], List of Prokaryotic Names with Standing in Nomenclature (LPSN) [17] and the NCBI’s Lifemap were used [18]. The details of the analyses were reported as follows: Concerning increased taxa, two studies showed no differences in the faecal samples [11,13]. The only similarities among the studies were an observed increase in the *Veillonella* genus [10] and the identification of *Veillonella* unclassified and *V. parvula* species in faecal samples [14].

Concerning the decreased bacterial taxa in MC compared to healthy controls, potential patterns were noticed. The most consistent among the studies was a decrease in the *Akkermansia* genus from the *Verrucomicrobia* phylum in the faecal samples [10,11,12,13]. In addition, the whole *Verrucomicrobia* phylum was described as decreased in [13]. A reduction in the *Coriobacteriaceae* family in both biopsy [8] and faecal samples [10] was described. Moreover, in the *Coriobacteriaceae* family, lower levels of *Coriobacteriaceae* unclassified [11] and *Collinsella* genus [10,11] were observed in the faecal samples. A decrease in the *Ruminococcaceae* [10] family; *Ruminococcaceae* NK4A214, UCG-002, UCG-005 and UCG-010 groups [12]; *Ruminococcaceae* unclassified [11]; and *Ruminococcus* 1 and 2 genus [12] in the faecal samples was described. Additionally, an increase in the *Ruminococcaceae* family and *Ruminococcus 2* genus was observed between baseline and after treatment with budesonide, which might indicate shifts in this taxa [12]. Furthermore, in the decreased *Rikenellaceae* family [10], a reduction in the *Alistipes* genus [11] and *Alistipes putredinis* species [14] was observed. Finally, *Clostridiales* unclassified [9,11], *Ruminiclostridium* genus [8], *Clostridium* genus [12] and *Clostridiales* unclassified [10] were decreased in the faecal samples. Whether there were actual changes for the mentioned taxa is uncertain.

Few studies also compared outcomes between MC and DC. However, no consistent results occurred. Similarities occurred with regards to the decreased OTUs from the *Ruminococcaceae* family in CD and UC [11] and an increased abundance of *Alistipes putredinis* in the functional DC [14]. Some taxa were described as increased or decreased in MC compared to the unspecified diarrhoea control [7]. One study reported no significant differences between the relative abundances in MC and the functional assessed together with bile acid DC [9].

In conclusion, there were observable common patterns for the increased and decreased taxa. More results are described in the “Additional Outcomes” column in Table 4.

## 4. Discussion

The analysis of the available data on the microbiome composition in MC in comparison to healthy controls poses the questions of whether there were actual differences among them and whether possible similarities existed between MC and other diarrhoeal diseases.

On a large scale, the changes in the alpha and beta diversities describe shifts in the microbiome composition. The findings differed among studies, showing either a lower alpha diversity compared to the healthy control [7,10,12] or no significant differences [8,9,11]. There are many possible variables that might influence the outcome. It was observed that the Shannon diversity index was significantly correlated with food and drink intake, physical activity, medication used, symptoms and blood clinical markers [19]. Therefore, the poor descriptions of the clinical details in the included studies might be a factor altering the conclusions. Nevertheless, the details regarding BMI indicated a wide diversity in patients. Therefore, the observed potential negative correlation between the Shannon index and both weight and BMI [19] might reveal a possible influencing factor on our findings. On the contrary, a recent meta-analysis showed no differences in the Shannon index in obese patients [20]. The relationship between obesity and alpha diversity might depend on the metabolic status of the individual, with a lower diversity among the unhealthy [21]. Therefore, the impact of BMI on the microbiome might not be straightforward. Not only BMI but also the appearance of diarrhoea in a week, number of bowel movements per week and abdominal pain were negatively correlated with alpha diversity status [19]. The stool’s form might be considered a possible factor influencing the assessment of the microbiome. Regarding CD and UC, a lower observed richness was detected in a recent meta-analysis [22]. Interestingly, one included study showed that the alpha diversity was similar for MC and CD/UC [10]. In another study, no differences between MC and functional DC or bile and acid DC were observed [9]. These findings support the question regarding similarities between MC and other diarrhoeal diseases. As for the beta diversity, the included studies showed no differences among the faecal samples [9] or biopsy samples [8]. In comparison, a lower beta diversity was observed in UC and CD compared to the non-IBD control, with a significant impact due to the samples’ type: faecal or biopsy [22].

Currently, the impact of the sample type on the microbiome assessment outcomes is being recognised as an important variable in studies. This might indicate the need to obtain biopsy samples for an analysis of microbiome composition instead of faecal samples due to the better representativeness of the colon microbiome [23]. On the contrary, emerging evidence shows that bowel preparation for a colonoscopy might not be without an influence on the outcomes of a microbiome assessment from biopsy [24] and faecal samples [24,25]. Moreover, the colonoscopy required for biopsy collection is an invasive procedure that is inadequate for healthy controls, with a small number of possible sampling sites and occurring concerns regarding contamination [26]. Here, included were two studies that used biopsy collection for a microbiome composition assessment [7,8]. Importantly, one of the studies used paraffin-embedded colonic tissue samples, which might be altered by the procedure [8]. It was difficult to conclude whether there were differences among the studies due to the small number of studies that were based on biopsy samples. Additionally, none of the studies described the time from the last diagnostic colonoscopy, except in [9]. Batista et al. [9] collected faecal samples three days before the diagnostic colonoscopy and 30 days after, thus limiting the impact of colonic cleansing. However, the faecal microbiome 30 days after a colonoscopy showed no changes in its composition [9]. Both the biopsy and faecal sampling might have influenced the outcomes of the included studies

The determination of the microbiome composition at a taxonomic level was beyond the scope of this review. However, possible patterns were recognised in the included studies that might be worth exploring. The most consistent result regarding taxa concerned a decrease in the *Akkermansia* genus from the *Verrucomicrobia* phylum in faecal samples [14,16,17,18]. Importantly, the presence of *Akkermansia* was observed in the healthy population [27]. Currently, the correlation between *Akkermansia* and obesity is being thoroughly examined. A lower level of *Akkermansia* was observed with obesity and associated with age and relative abundance. However, the correlation between *Akkermansia* and being overweight was insignificant [28]. Here, a description of BMI was present in only one of the studies, where BMI ranged from 17 to 35 [11]. Therefore, patients with obesity were included in the study population. Interestingly, in the study by Carstens et al. [11], the impact of BMI was proved to be significant between participants with BMI < 30 and >30, but no differences occurred between the study and control populations concerning BMI. As no other study described BMI, it should be taken into consideration as a variable that might influence the level of *Akkermansia*. In comparison with other gastrointestinal diseases, a decrease in *Akkermansia* was also observed in a meta-analysis of UC [22]. It might be crucial that the *A. muciniphila* species belongs to mucin-degrading bacteria [29,30]. Therefore, it is involved in the maintenance of the mucus layer and might play a role in gut homeostasis. Whether *Akkermansia* and its species have a potential role in colitis is the subject of ongoing, robust studies.

The *Veillonella* genus is known to be a part of a healthy oral microbiome [31]. Moreover, *Veillonella* was also associated with a healthy microbiome detected in faecal samples [32], but no conclusion on its oral or colon origin could be made due to the impossibility of their detection at the species level [27]. In general, stool microbiota differs from upper tract microbiota [27]. The link between the oral and gut microbiomes is yet to be determined [27,33]. There is a hypothesis that considers an influence through bacterial or immune pathways [33]. As PPI administration is a risk factor for MC, it might be worth noticing that PPI was connected with a higher prevalence of oral cavity bacteria in the gastrointestinal microbiome [34]. Even though *Veillonella* was recognised in a healthy population, a meta-analysis described an increased abundance of *Veillonella* in CD and UC [22]. Therefore, it might be interesting that *Veillonella* and *Veillonella* unclassified were reported to be increased in faecal samples in [10] and [14], respectively. At the species level *V. parvula* was detected to be increased in faecal samples [14]. Recently, it was also described as elevated in faecal samples from CD [35] and associated with the inflammatory potential of diet in CD [36]. Whether there is an actual connection between *Veillonella* and gut inflammation might be a perspective for future studies.

Microbiome changes might imply the relevance of therapy that focuses on the gut bacteria through the administration of probiotics. Currently, the administration of probiotics in CD and UC is controversial. The American Gastroenterology Association (AGA) only supports the use of probiotics in clinical trials [37]. In comparison, the European Crohn’s and Colitis Organisation (ECCO), in a review on complementary medicine and psychotherapy in IBD, concluded that there is not enough evidence for the use of probiotics in CD, but *Escherichia coli* Nissle 1917 or multi-strain probiotics containing a combination of lactic acid bacteria, *Streptococcus* and *Bifidobacteria* might be considered as an alternative for the induction and maintenance of remission in UC [38]. Large RDBCTs with follow-up are required to determine the influence of probiotics in MC. Interestingly, engineered probiotics have recently been investigated in mouse models for the treatment of IBD [39]. These probiotics might be the future for treatments related to the microbiome.

There were several limitations to this study. First, no synthesis in a meta-analysis format was possible. Second, the descriptions of the study population and clinical features were poor. Therefore, the detection of some important factors with a potential influence on the outcomes was difficult. Furthermore, various exclusion and inclusion criteria were also used for the study population. Therefore, this might have interfered with the comparison of the studies. Finally, the descriptions of the taxonomy levels varied among the studies, which sometimes made it difficult to compare the results. Therefore, no specific pathogenic species were determined. In conclusion, our findings might be considered as an overview and guide for future studies with a strong focus on microbiome composition in patients suffering from MC.

## 5. Conclusions and Future Directions

The microbiome composition is potentially altered in MC; however, no firm agreement on the composition or taxa related to the pathogenesis or course of MC can be made. Future studies could focus on factors that influence the composition of the microbiome. It might be relevant to establish the relationship between the risk factors of MC and the microbiome, which might provide better insight into the pathogenesis of MC. Further research regarding the correlation between MC and other gastrointestinal diseases might also be important for a better understanding of MC. Moreover, it might be important to determine the pathogenic species for MC. Consequently, such results might become a guide for the treatment and guidance of patient.

## Figures and Tables

**Table 1:** Description of the included studies concerning microscopic colitis.

Study	Year	Country	Study Design	Study Population (*n* = 183)	Control Population (*n*= 444)	Newcastle–Ottawa Scale
Sun S. [7]	2022	USA	CCS	53	Unspecified diarrhoea (*n =* 153)	6
Millien V. [8]	2018	USA	CCS	20	Healthy (*n =* 20)	6
Batista L. [9]	2022	Spain	CCS	16	Healthy (*n =* 14) Bile acid diarrhoea (*n =* 16) Functional diarrhoea (*n =* 11)	6
Hertz S. [10]	2021	Denmark	CCS	15	Healthy (*n =* 21) Crohn’s disease (*n =* 21) Ulcerative colitis (*n =* 38)	6
Carstens A. [11]	2019	Sweden	CCS	29	Healthy (*n =* 29) Crohn’s disease (*n =* 32) Ulcerative colitis (*n =* 32)	7
Krogsgaard L.R. [12]	2019	Denmark	CCS	20	Healthy (*n =* 10)	6
Fischer H. [13]	2015	Sweden	CCS	10	Healthy (*n =* 7)	6
Morgan D.M. [14]	2020	Sweden	CCS	20	Healthy (*n =* 20) Functional diarrhoea (*n =* 20)	6

CCS, case-control study.

**Table 2:** Description of the study populations suffering from microscopic colitis: microbiome *(n* = 183).

Study	Age (Mean ± SD)	Sex (% Female)	BMI (Mean ± SD)	Autoimmune Disorder (%)	Budesonide (% Treated)	Smoking (%)	PPI (% Treated)	NSAID (% Treated)	Antibiotics Elimination before Inclusion (Month)	Probiotic Elimination before Inclusion (Month)	Other Medication
Sun S. [7]	62.2 ± 13.5	90.4	25.2 ± 6.2	N/A	N/A	N/A	N/A	N/A	3	N/A	N/A
Millien V. [8]	62 Range: 27–83	5	N/A	N/A	N/A	25	50 H2RA: 10	50	3	N/A	N/A
Batista L. [9]	61.8 ± 2.9 ^1^	81	24.7 ± 1.2	N/A	N/A	N/A	N/A	18,7	3	3	Elimination of herbal remedies 3 months prior
Hertz S. [10]	Median: 72	74	N/A	N/A	N/A	N/A	N/A	N/A	1	N/A	N/A
Carstens A. [11]	Median: 65/64 ^2^ Range: 33–89/33–82 ^2^	72	Median 25 Range 17–35	21	Oral corticosteroids: 59	N/A	N/A	N/A	3	N/A	N/A
Krogsgaard L.R. [12]	Median: 67.0 IQR: 55.3–71.8	70	N/A	N/A	100	Current 30	20	No, 1 month prior	3	1	N/A
Fischer H. [13]	48, Range: 43–68 50, Range: 43–65	100	N/A	N/A	N/A	27	N/A	N/A	N/A	N/A	N/A
Morgan D.M. [14]	63.2 ± 8.5	80	24,7 ± 3,5	N/A	N/A	Current: 20 Previous: 25 Never: 55	N/A	N/A	N/A	N/A	N/A

^1^ Inclusion criteria: women ≥50 years and men ≥70 years. ^2^ The first values were derived from the text and the second values from the table regarding the baseline demographics. BMI, body mass index; PPI, proton pump inhibitor; NSAID, nonsteroidal anti-inflammatory drug; H2RA, histamine 2 receptor antagonists.

**Table 3:** Description of microscopic colitis.

Study	Subtype (%)	Diagnosis Verification (Biopsy)	Duration of Disease (Months)	Clinical Activity (Hjorstwang) (% Active)	Diarrhoea Characteristics	Daily Stools Number
Sun S. [7]	N/A	Yes	N/A	N/A	N/A	N/A
Millien V. [8]	CC: 55; LC: 45	Yes	N/A	N/A	Duration: <12 months, 40%; >12 months, 60%	<3: 45%; 3–5: 20%; >5: 35%
Batista L. [9]	N/A	Yes	N/A	N/A	Duration: Median: 20; IQR: 8–156 weeks	Total: Median: 5; IQR: 3–7.5 Liquid: Median: 4.5; IQR: 2–5.5
Hertz S. [10]	CC: 47; LC: 53	No	N/A	Assessed as diarrhoea ≥ 3 stools/day: 100	N/A	N/A
Carstens A. [11]	CC: 100	No	N/A	34	N/A	N/A
Krogsgaard L.R. [12]	CC: 50; LC: 50	No	N/A	Baseline: 100; 8 weeks: 40; 16 weeks: 50	N/A	N/A
Fischer H. [13]	N/A	No	N/A	N/A	N/A	N/A
Morgan D.M. [14]	N/A	No	N/A	Assessment in the active (≥3 bowel movements; ≥5 Bristol) and remission phases	N/A	N/A

CC, collagenous colitis; LC, lymphocytic colitis.

**Table 4:** Results of the microbiome assessment in patients with microscopic colitis.

Study	Type of Samples	Method	Microbiome Composition in MC Compared to HC			Microbiome Composition in MC Compared to DC	Additional Outcomes
			Increased	Decreased	Indexes		
Sun S. [7]	Biopsy samples: fresh frozen (colon: ascending, descending)	Amplification: 16S rRNA, V2 region Sequencing: Illumina MiSeq	No healthy control.			Shannon index was lower for MC model 2 and 4 ^1^ for biopsy from ascending colon and for all 4 modes ^1^ for biopsy from descending colon (*p* < 0.05). Increased (*p* < 0.1) *Betaproteobacteriales*, *Burkholderiaceae* *Haemophilus* unclassified and *Streptococcus* unclassified ^1^ Decreased (*p* < 0.1) *Coriobacteriales* *Coriobacteriia* ^1^.	Microbiome composition at the genus-level was significant only for samples from descending colon (*p* = 0.043).
Millien V. [8]	Biopsy samples: obtained from tissue bank as formalin-fixed and paraffin-embedded tissues	16S rRNA primers, V4 region sequencing	OTU (*p* < 0.05): *Desulfovibrio* *Desulfovibrionales* *Desulfovibrionaceae* *Sphingobacteriia* *Sphingobacteriales* *Deltaproteobacteria* *Rhodobacterales* *Rhodobacteraceae* *Ruminiclostridium* *Lachnospiraceae*	OTU (*p* < 0.05): *Bacillales* *Enterobacter* *Staphylococcaceae* *Corynebacteriales* *Lawsonella* *Subdoligranulum* *Prevotella* *Coriobacteriaceae* *Coriobacteriia* *Coriobacteriales* *Propionibacteriaceae* *Propionibacterium*	Shannon index and beta diversity: no differences (influence of PPI or NSAIDs was also assessed as insignificant).	No	*Actinomyces* and *Faecalibacterium* abundance was higher in MC not taking PPI than in MC on PPI. Bacilli abundance was increased in MC on NSAIDs compared to MC not taking NSAIDs.
Batista L. [9]	Faecal samples collected before diagnostic colonoscopy	Amplification: PCR, 16S rRNA, region V4 Sequencing: Illumina	OTU (*p* < 0.05) (no FDR): *Clostridium perfringens* *Clostridium saccharogumia* *Dialister* sp. *Veillonella parvula* *Citrobacter* sp.	OTU (*p* < 0.05) (FDR): *Clostridiales* unclassified OTU (*p* < 0.01) (no FDR): *Bacteroides* *Bifidobacterium adolescentis* *Lachnospiraceae* unclassified *Coprococcus* *Ruminococcaceae* unclassified *Ruminococus* *Catenibacterium* OTU (*p* < 0.05): *Coriobacteriaceae* *Bacteroidaceae* *Porphyromonadaceae* *Rikenellaceae* *Clostridiaceae* *Erysipelotrichaceae* *Acidaminococcaceae* *Succinivibrionaceae*	Shannon index and beta diversity: no differences.	Shannon index was lower in MC and functional + bile acid DC compared to HC. No differences between MC and DC. Beta diversity: no differences. Microbial dysbiosis index ^3^ was higher in MC than functional and bile acid DC (*p* = 0.014). Bacterial composition: no differences.	No differences between CC and LC for alpha diversity, beta diversity and microbial dysbiosis index were observed. Increase in *Lachnospira* (*p* < 0.03) was observed 30 days after PEG colonic cleansing.
Hertz S. [10]	Faecal samples	Amplification: PCR, 16S rRNA, V4 region Sequencing: Illumina NextSeq 500	OTU (*p* ≤ 0.1): *Prevotella copri*, *P. stercorea* *Veillonella* *Gammaproteobacteria* *Roseburia* *Enterobactericeae* *Streptococcus* *Odoribacter* *Fusobacterium* *Erysipelotrichaceae* *Aggregatibacter*	OTU (*p* ≤ 0.1): *Blautia* *Dialister* *Butyricimonas* *Clostridiales* *Ruminococcaceae* *Sutterella* *Akkermansia* *Parabacteroides* *Rikenellaceae* *Oscillospira* RF-32 *Desulfovibrionaceae* *Coriobacteriacea* *Bacteroidales* *Christensenellaceae* *Anaerostipes* *Anaeroplasmataceae* *Adlercreutzia* *Mogibacteriaceae* *Collinsella*	Alpha diversity (*p* < 0.01) and taxonomic richness (*p* = 0.0003) were lower in MC.	Alpha diversity in MC comparable to CD and UC (*p* > 0.05).	No differences in the alpha diversity between LC and CC. *Prevotella* dominated MC microbiome in comparison to HC (*p* = 0.03).
Carstens A. [11]	Faecal samples	Amplification: PCR, 16S rRNA, V3-V4 region Sequencing: Roche 454-FLX GS100	No differences	OTU (*p* < 0.05): *Collinsella* *Methanobrevibacter* *Coriobacteriaceae* unclassified *Desulfovibrio* *Halomonas* *Acetivibrio* *Ruminococcaceae* unclassified *Clostridiales* unclassified *Erysipelotrichaceae incertae sedis* *Turicibacter* *Erysipelotrichaceae* unclassified *Alistipes* *Akkermansia*	Shannon diversity index: no difference	Similarly to active CC (assessed together with the population on steroid treatment), some OTUs related with *Ruminococcaceae* family also decreased in CD and UC (*p* < 0.05).	Active CC (assessed together with population on steroid treatment) was associated with decreased abundance of *Collinsella*, *Ruminococcaceae* unclassified, *Clostridiales* unclassified and *Coriobacteriaceae* unclassified (*p* < 0.05).
Krogsgaard L.R. [12]	Faecal samples	Amplification: 16S rDNA, V3-V4 region 18S rDNA primers, V9 region Sequencing: Illumina MiSeq	OTU (*p* < 0.05): Baseline CC: *Faecalibacterium* Baseline LC: *Sutterella* During treatment MC: *Ruminococcaceae* and *Ruminococcus 2*	OTU (*p* < 0.05): Baseline CC: *Akkermansia*, *Anaerotruncus*, *Chritensenellaceae* R-7 group, *Clostridium sensu stricto*, *Coprococcus*, *Romboutsia*, *Ruminococcus 1* and *2*, *Ruminiclostridium*, *Ruminococcaceae* NK4A214, UCG-002, UCG-005, UCG-010 group Baseline LC: *Romboutsia Ruminococcaceae* NK4A214 group During treatment MC: *Faecalibacterium*	Alpha diversity was lower in LC and CC at baseline and increased during treatment (16 weeks: *p* < 0.001).	No	There were no differences in bacterial abundance between LC and CC. In both LC and CC, >50% of microbiome consisted of *Faecalibacterium*, *Bacterioides*, *Prevotella* and *Blautia*, whereas the microbiome composition of HC was more balanced.
Fischer H. [13]	Faecal samples	Metagenomic sequencing: Illumina HiSeq 2000	No differences	Abundance (*p* = 0.02): *Veruccomicrobia* (*Akkermansia* spp.)	N/A	No	Level of *Akkermansia muciniphila* (log CFU) was lower in MC who were smokers. ^2^
Morgan D.M. [14]	Faecal samples	Shotgun metagenomics, sequencing: Illumina HiSeq2500	OTU (*p* < 0.1): *Haemophilus parainfluenza* *Veillonella parvula* *Veillonella* unclassified	OTU (*p* < 0.1): *Alistipes putredinis*	Microbial dysbiosis index ^3^ was higher in active MC than in remission MC or HC.	Abundance of *Alistipes putredinis* increased in functional DC (q = 0.043). Microbial dysbiosis index was higher in active MC than in functional DC.	No differences at the species level for active and remission MC. Alpha diversity was lower in active than in remission MC (q = 0.031). No other differences. Increase in global PTR (q = 0.033) and *Alistipes finegoldii*-specific PTR (q = 0.065) were observed in active MC compared to HC.

^1^ Microbiome of MC was compared to microbiome in HC as four different models adjusted for the population’s features (1: education, proton pump inhibitor use and batch; 2: 1 + sex and antibiotics use; 3: 2 + age; 4: 3 + BMI). Only the outcomes that were in common for models 1, 2 and 3 or 1, 2, 3 and 4 are included in Table 4. ^2^ The level of *Akkermansia* in smokers was assessed in larger groups than those included in the study; an additional 5 female with MC and 7 HC were included for the PCR analysis. ^3^ Log of (total abundance in organisms increased) over (total abundance of organisms decreased). FDR, false discovery rate; MC, microscopic colitis patients; HC, healthy control; DC, diarrhoea control; CC, collagenous colitis; LC, lymphocytic colitis; FD, functional diarrhoea; CD, Crohn’s disease; UC, ulcerative colitis; NSAIDs, nonsteroidal anti-inflammatory drugs; PPI, proton pump inhibitor.

## Data Availability

No additional data were created during the analysis.
